# Supplementary material for: Patterns of Intron Gain and Loss in Fungi
Source: PLoS Biol. 2004 Nov 30;2(12):e422. doi: 10.1371/journal.pbio.0020422 (PMC532390; doi:10.1371/journal.pbio.0020422)
Supplement: Table S1 — Also available at http://genes.mit.edu/NielsenEtAl/. (4.3 MB ZIP). [file pbio.0020422.st001.zip › NielsenEtAl/html/64.html]

AN0243.1.NCU07320.1.MG04947.1.FG04985.1


```
 CLUSTAL W (1.82) Multiple Sequence Alignments - Introns Inserted


Sequence 1: NCU07320.1	400 aa
Sequence 2: MG04947.1	508 aa
Sequence 3: FG04985.1	424 aa
Sequence 4: AN0243.1	444 aa
Alignment Length: 516 aa
Number Identitical Residues: 146 aa
Alignment Score (without introns) 7722


MG04947.1 	MC0HWGVGLQSKPTGPLAAGKVGFSNGSVNTFTVAAGTAQAIVVCLAQVHE2YQTRPLLF
NCU07320.1	--~------------------------------------------------~--------
FG04985.1 	--~------------------------------------------------~--------
AN0243.1  	--~------------------------------------------------~---MPLWR
          	                                                        .   

MG04947.1 	LISSLRSPAKPRSFHFNKRIAPRMTGSTEYATGATADPEVGYPKGHLGHLTDSETKALSE
NCU07320.1	-----------------------MTADSIADASAASGTEVGYPNGHLGHLKPEEQEALES
FG04985.1 	MAANLEAPG---------------APAERITSTASTIADYAYPYGHLGHLTQKQEEAFVQ
AN0243.1  	TKSHHSQAESDSASVQNKADTTPADPKNDPVASASQAASDAWLVGHLNHLTEEQEAKLTE
          	  :    . .  :   ..  :.         : *:  .. .:  ***.**. .:   : .

MG04947.1 	FKVLVTENGLYTPG---PPPSHDDQTLL2RFLRARRWVPQDAFKQFKETEEWRSTTRLDT
NCU07320.1	FKTNLAEKGYYKPG---PPASHDDQTLL2RYLRARRWNVVDAFKQFKETEDWRKANDLNV
FG04985.1 	FKKVLEERGLLTP-------AHDDPLIL2RYLRARRWNVEDAYTQFKETEDWRKANDLNV
AN0243.1  	FKAFCEKEGYYKPGGEGEKPSHDDATML2RFLRARRFDVNGAWGQFKDTEDWRKENAIEA
          	**    :.*  .*....  .:***  :* *:*****:   .*: ***:**:**. . ::.

MG04947.1 	LYDTIELDSYEQSRVM0YPQWTGRRDKR~GIPVYLFEIKQLDSKAVAAYEKG-ADDTY-S
NCU07320.1	LYDTIDLEAYEASRRL~YPQWTGRRDRR2GIPLYLFEIRHLDSKTVSAYEKA-AETNP-S
FG04985.1 	LYDTIDLSAYDFSRRL0YPQWTGRRDRR~GIPLYVFEVKTLDSKTVHEYEKVGASSTF-S
AN0243.1  	LYENIDVDSYEAARRM0YPQWTGRRDRR~GIPVYVFEIKHLNSKNMAAYNATMADPEATA
          	**:.*::.:*: :* : *********:* ***:*:**:: *:** :  *:   *.   ::

MG04947.1 	RAHADGSTPPKLLRLFALYENLTRFAQPLCTEMPDRPHP-QTPITLSTNIVDVSGVSLRQ
NCU07320.1	KAVTDGQTSPKLLRLFALYENLTRFAQPLCTELPDRPHATTTPITLSTNIVDVSGVSLRQ
FG04985.1 	QAKSDGKTPNGLLRLFALYENLTRFNMPFCTQLLDRDHP-EVPITLSTNIVDISGVGLKQ
AN0243.1  	ETHKSSAVPQRLLRLFALYENLLNFVMPLCSELP-RPHP-ETPIVSSNNIVDVSGVGLKQ
          	.:  .. ..  *********** .*  *:*:::  * *.  .**. *.****:***.*:*

MG04947.1 	FWNLKAHMQAASTLATAHYPETLDRIF~VIGAPFFFSTVWGWIKRWFDPITVSKIFILSQ
NCU07320.1	FWNLKSHMQAASQLATAHYPETLDRIF~IIGAPYFFSTVWGWIKRWFDPITVSKIFILGP
FG04985.1 	FWNLKQHMQAASQLATAHYPETLDRIF~VIGAPAFFSTVWGWVKRWFDPITVSKIFILGS
AN0243.1  	FWNLKGHMQDASVLATAHYPETLDRIF0IIGAPAFFPTVWGWIKRWFDPVTTSKIFILSA
          	***** *** ** ************** :**** **.*****:******:*.******. 

MG04947.1 	AEVLPVLSSFMEVENIPKQYGGKLEFKWCDQPNLDPAIKERA-TWEQGFDNFPKGPVLWK
NCU07320.1	SEVKATLEEFIDPKNIPKQYGGELDFTWGDQPKTDPYIKETV-KWENGLKDFPEGPKYWR
FG04985.1 	HEVKTVLEQYIEPRNIPKKYGGELDYNFGELGNPDPNWQGVV-EFENGHKTFPSGPLLWE
AN0243.1  	SEVLPTLTSFMEPSSIPKQYGGELDWQWGDMPNLDEPARELIGALETAPADGQTKPGFIK
          	 ** ..* .:::  .***:***:*:: : :  : *   :   .  * .       *   .

MG04947.1 	-VLDEERLECVAVGSVNQKQRNIRVCTLPRSFKGVVSSNQPYHKTEAAAAASDIAAVETG
NCU07320.1	PTADGTRMECVAVGSEGGKQRMLSVGTLERVCN---------------VKKVDGDALAEG
FG04985.1 	-EDGNDRLVCVAKGSKDGKPRNERICTIPKTFG---LSPVVEIGGHAPVDAAPVNGVADG
AN0243.1  	GPVLFKGDKVEVLGKENGKSRATTVPVPESKLA-----------QTNGVSATNQSEKEQE
          	           . *. . * *   : .                     .           

MG04947.1 	VQKLDIQEAKSDSEAVTSSKEEIIAEKGASSVDAPAASAKAA
NCU07320.1	VKGLSVGDAEKN-EIVETGKEATVPTVSA-------------
FG04985.1 	TAGAEGAQTDDSTLSGEVNEKLKIQEDKPAVPQTTAA-----
AN0243.1  	LQPAAEKEQEVSEKVFGNDTAAKTNGDVAPPTQLSA------
          	       : . .      .         ..  . .:
```
